# Novel Amides Derivative with Antimicrobial Activity of *Piper betle* var. *nigra* Leaves from Indonesia

**DOI:** 10.3390/molecules26020335

**Published:** 2021-01-11

**Authors:** Fajar Prasetya, Supriatno Salam, Agung Rahmadani, Kansy Haikal, Lizma Febrina, Hady Anshory, Muhammad Arifuddin, Vita Olivia Siregar, Angga Cipta Narsa, Herman Herman, Islamudin Ahmad, Niken Indriyanti, Arsyik Ibrahim, Rolan Rusli, Laode Rijai, Hadi Kuncoro

**Affiliations:** 1 Faculty of Pharmacy, Universitas Mulawarman, Samarinda 75123, Kalimantan Timur, Indonesia; fajarprasetya@farmasi.unmul.ac.id (F.P.); agungchempharm8794@gmail.com (A.R.); lizmafebrina@farmasi.unmul.ac.id (L.F.); marifuddin@farmasi.unmul.ac.id (M.A.); vitaoliviasiregar@farmasi.unmul.ac.id (V.O.S.); angga@farmasi.unmul.ac.id (A.C.N.); herman@farmasi.unmul.ac.id (H.H.); islamudinahmad@farmasi.unmul.ac.id (I.A.); nikenindriyanti@farmasi.unmul.ac.id (N.I.); arsyik@farmasi.unmul.ac.id (A.I.); rolan@farmasi.unmul.ac.id (R.R.); laoderijai@farmasi.unmul.ac.id (L.R.); 2 Department of Chemistry, Faculty of Mathematics and Natural Sciences, Universitas Padjadjaran, Jatinangor, Sumedang 45363, Indonesia; supriatno.salam@gmail.com (S.S.); kansyhaikal@unpad.ac.id (K.H.); 3 Departement of Chemistry Education, Faculty of Teaching and Education, Mulawarman University, Samarinda 75123, Kalimantan Timur, Indonesia; 4 Departement of Pharmacy, Faculty of Mathematics and Natural Sciences, Islamic University of Indonesia, Jogjakarta 55584, Indonesia; hadyanshory@uii.ac.id

**Keywords:** *Piper betle* var. *nigra*, *Streptococcus mutans*, *Streptococcus sanguinis*, *Candida albicans*, piperenamide A, piperenamide B

## Abstract

*Piper betle* var. *nigra* is a tropical plant closely related to the common piper. *P. betle* has also been dubbed a promising source of natural antioxidants in herbal health products, antibacterial, antifungal, antimalarial, cytotoxic activity against the cancer cell lines K562 and HL-60, and antileishmanial. The aim of this study to observation Antimicrobial activity and isolation of chemical compound. The antimicrobial activity of *P. betle* extract was performed by well diffusion method against two oral pathogenic bacteria (*Streptococcus mutans* and *Streptococcus sanguinis*) and opportunistic pathogenic yeast (*Candida albicans*). The inoculum (bacterial and yeast suspension) was prepared from a 24-h culture on NB for bacterial suspension and on TSB for yeast suspension. Extraction and isolation using various method of chromatography. Isolated compounds were characterized by spectroscopic means. Our study showed antimicrobial activity from crude ethanol extract of leaves *P. betle* L. var. *nigra* against two oral pathogenic bacteria and opportunistic pathogenic yeast with concentration 0.5% and 1%. The first report of two new amides derivatives, piperenamide A (1) and piperenamide B (2) in *P. betle* L. var. *nigra*.

## 1. Introduction

*Piper betle* a piper species have a simple profile contain very diverse suites of secondary metabolites and responsible for their use in traditional medicines to treat several disease [1]. *Piper betle* L. var. *nigra* or black betle (in Indonesia known as Sirih Hitam) is a tropical plant closely related to the common piper and belongs to the Piperaceae family and the genus of piper. This genus consists of five subgenera and approximately 1400 species spread throughout tropical and subtropical regions and widely cultivated in Indonesia, India, Sri Lanka, Malaysia, Thailand, Taiwan, and other Southeast Asian countries and has a long history of over 2000 years. This plant use for decoration and medicinal plants. *P. betle* is one of the most potent medicinal herbs that has been used over the years. In addition to the large number of beneficial properties, *P. betle* has also been dubbed a promising source of natural antioxidants in herbal health products, antibacterial, antifungal, antimalarial, cytotoxic activity against the cancer cell lines K562 and HL-60, and antileishmanial [2,3,4,5,6,7,8]. Chemical composition from *Piper betle* L. var. *nigra* included caryophyllene, cadinene, γ-lactone, allyl catechol, p-cymene, eugenol methyl ether, 4-allyl resorcinol, stigmast-4-en-3,6-dione, and aristololactam A-II, and essential oils such as chavicol, chavibetol, chavabetyl acetate, eugenol, eugenyl acetate, safrole, (E) Isoeugenol, and B-caryophyllen, pipercerebrosides A and B, and amides alkaloid [8,9,10]. Amides as a class of typical constituents. More than 300 members of amide alkaloids are already found in species of the genus Piper. It seems most of them have possible bioactivity, such as antifungal, antiepileptic, antidepressant, hepatoprotective, and antiplatelet aggregation activities [11]. Isobutyl amides are one of the most frequently known classes o the amides in the plants. These amides are primarily found as long chain conjugates in piper genus. Arboreumine, pellitorine, fagaramide, brachystamides-C, D, E, retrofractamide-D, N-isobutyl-4-hexanoyl-hydroxypyrrolidin-1-one, (±)-threo-N-isobutyl-4,5-dihydroxy-2E-octaenamide, scutifoliamide A, B, hoffmannseggiamide A, B, and cyclopipperettine [12,13].

In this study we reported the isolation and elucidation of the chemical structures two novel amide derivatives compound and evaluated the antimicrobial activity effect of the crude ethanol extract of *Piper betle* var. *nigra* leaves from Indonesia. Antimicrobial assay to be carried out for the activity of ethanol extract from *P. betel* leaves against the activities of antimicrobial against two oral pathogenic bacteria (*Streptococcus mutans* and *Streptococcus sanguinis*) and opportunistic pathogenic yeast (*Candida albicans*).

## 2. Results and Discussion

### 2.1. Antimicrobial Activity

**Figure 1:**
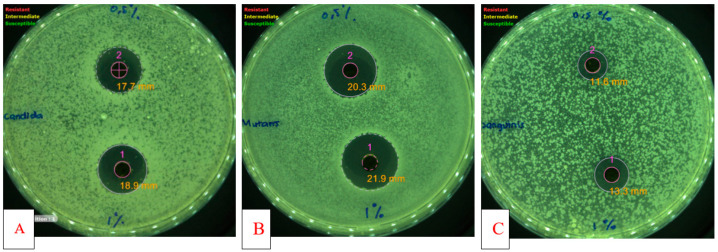
Antimicrobial activity of *Piper betle* var. *nigra* extract. (**A**) Antifungal activity against *Candida albicans*; (**B**) Antibacterial activity against *Streptococcus mutans*; (**C**) Antibacterial activity against *Streptococcus sanguinis.* 1 (concentration of 1%); 2 (concentration of 0.5%).

Table 1 shows the antibacterial activity of *P. betle* extract against two oral pathogenic bacteria and an oral opportunistic fungal. *P. betle* extract in all concentration from 0.5% to 1% inhibited growth of *S. mutans, S. sanguinis,* and *C. albicans* with increasing diameters of inhibition observed with increasing concentration of *P. betle* extract (Figure 1). *S. mutans* was the most sensitive strain compared to *S. sanguinis* and *C. albicans* against *P. betle* extract (0.5%) with inhibition diameters of 18.2 mm, 9.9mm, and 16.7 mm, respectively. The difference in sensitivity of these organisms is probably due to different cell types. Candida (yeast) cell types are different from streptococci (bacteria). This causes differences in sensitivity to *P. betle* extract. Sensitivity of *S. mutans* are more susceptible than *S*. *sanguinis.* The possible causes are many factors, including differences in virulence. In 2015, Azizi et al. assed the sensitivity of *S. mutans* and *S. sanguinis* to *Zingiber officinale*. They showed that *S. mutans* more susceptible than *S. sanguinis* with MIC value of 0.02 and 0.3 mg/mL, respectively [14]. In addition, their sensitivity to chlorhexidine were also reported by Medina-Flores et al. (2016), where *S. mutans* more susceptible than *S. sanguinis* with growth inhibition 23.97 mm and 19.80 mm, respectively. Alkaloid, terpenoid, flavonoid, polyphenols, tannin, and saponin compounds have been identified from *P. betle* leaf extract [15]. Flavonoids, polyphenols, and tannins are known to have antibacterial activity with at least five possible mechanisms: damage the cell membrane permeability, inhibit protein synthesis, damage the bacterial cell wall, inhibit ATP synthesis, and interfere with cell [16]. The antibacterial activity that we obtained is thought to be derived from the activity of the isolate compounds of Piperamide A and B was isolated from *Piper betle* var. *nigra*. This is as has been reported from other plants of the Piper genus because of the similarity in structure to the compounds we obtained although further testing is needed to ensure the strength of the antibacterial activity of these compounds [17,18,19]. Table 2 shows none of the tested samples of Piperamide A and B showed antimicrobial activity against *S. mutans, S. sanguinis,* and *C. albicans* up to 0.02%. These result from amides derivative showed the same results in tests for cyclopipperetine on the Piper nigrum [13]. In recent years, *Candida* species has demonstrated resistance to many synthetic medications, indicating the need for new antifungal drugs with less side effects to treat candidiasis effectively. Several experiments used natural substances against multiresistant strains and in particular against azole resistant candidiasis have indicated that certain species of plants have promising antimicrobial compounds from natural substances [20,21,22].

### 2.2. Isolation of Crude Ethanol Extracf of Piper betle var. nigra

The EtOH extract from the leaf of *P. betle* L. var. *nigra* was chromatographed over a vacuum-liquid chromatographed (VLC) column, packed with silica gel 60 by gradient elution. The VLC fractions were repeatedly subjected to normal and reverse phase column chromatography, as well as preparative TLC on silica gel GF_254_ to accommodate compounds **1**–**2**.

**Figure 2:**
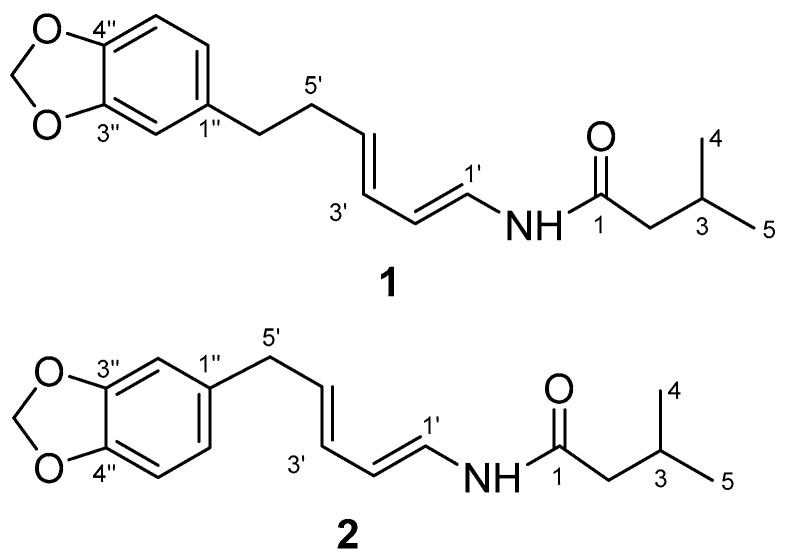
Structures of piperenamide A-B (**1**–**2**).

**Figure 3:**
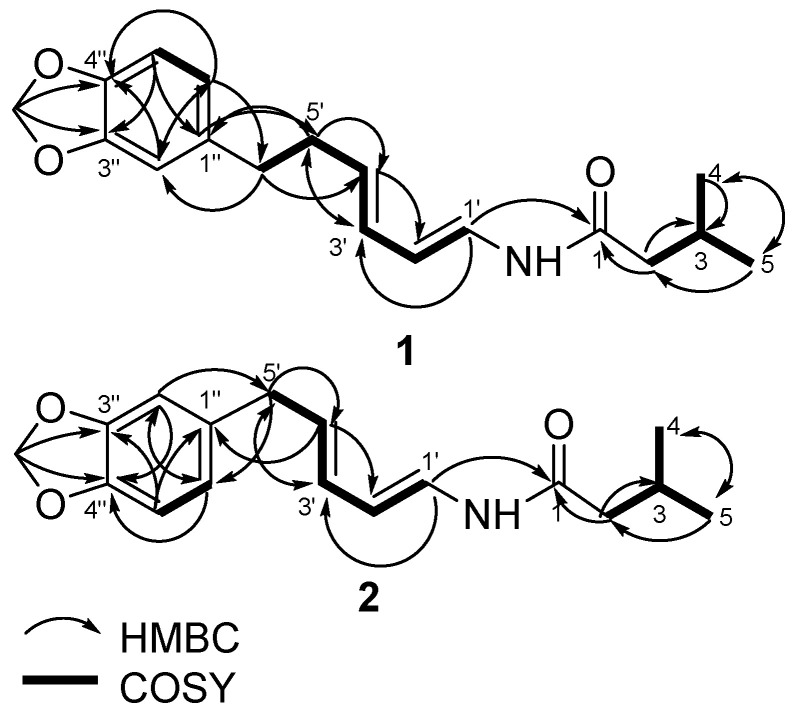
Selected HMBC and COSY correlations for piperenamide A-B (**1**–**2**).

Piperenamide A (**1**) was observed as a colorless amorphous solid, with its molecular composition established as C_18_H_23_NO_3_, based on HR-TOFMS. This showed a [M + H]^+^ ion peak at **m*/*z** 302.1749 (calcd. C_18_H_24_NO_3_ **m*/*z** 302.1756), requiring to eight degrees of unsaturation (Figure 2). The UV spectrum showed maximum absorption at 270 and 241 nm, indicating the presence of a benzene and conjugated alkene. The IR spectrum showed bands which were ascribed to an amine (ν_max_ 3295 cm^−1^), amide carbonyl (ν_max_ 1656 cm^−1^), isolated double bond conjugated (ν_max_ 1611 and 1504 cm^−1^), and ether groups (ν_max_ 1241 cm^−1^). Furthermore, the ^1^H-NMR spectrum (Table 3) showed two primary methyls at δ_H_ 0.90 (6H, d, 6.7 Hz, Me-4 and 5), four sp2 methine protons at δ_H_ 5.92 (1H, dd, *J* = 4.6; 15.1 Hz, H-1′), 7.02 (1H, dd, *J* = 10.6; 15.1 Hz, H-2′), 6.09 (1H, dd, 10.6; 15.2 Hz, H-3′), and 6.18 (1H, dd, 7.2; 15.2 Hz, H-4′), suggested the presence of an α,β,γ,δ-unsaturated secondary amide, two trans double bonds (*J*1′,2′ = 15.1 Hz, *J*_3′,4′_ = 15.2 Hz), one cis double bonds (*J*_2′,3′_ = 10.6 Hz), tri-substituted benzene at δ_H_ 6.63 (1H, dd, *J* = 1.5; 7.9 Hz, *m*; o, H-6″), 6.69 (1H, d, 1.5 Hz, *m*, H-2″), and 6.69 (1H, d, 7.9 Hz, *m*, H-5″), a methylene dioxyphenyl δ_H_ 5.88 (2H, s) and some aliphatic signals in the upfield region (Supplementary Materials). Comparison of the NMR data of 1 with those of guineensine [23] shows the same amide chain but with different N-substituent positions. The N-substituent of 1 position is attached to the olefin proton resonances, this is indicated δ_H_ 3.06 (2H, d, 6.7 Hz, H-2) no correlation to δ_H_ 4.62 (NH s), δ_H_ 5.92 (1H, dd, *J* = 4.6; 15.1 Hz, H-1′) and guineensine position is attached to the iso-butyl, this is indicated δ_H_ 3.16 (2H, t, 6.4; 12.9 Hz, H-1′) correlation to δ_H_ 5.60 (NH brs), δ_H_ 5.76 (1H, d, 15.0 Hz, H-2). The ^13^C NMR together with the DEPT spectra revealed eighteen carbons consisting of an amide carbonyl at δC 169.1, α,β,γ,δ-unsaturated secondary amide at δ_C_ 123.3, 141.9, 142.8 and 130.2, tri-substituted benzene at δ_C_ 136.5, 109.7, 149.0, 147.9, 147.9, 108.8, and 122.3, carbon methylene dioxyphenyl at δ_C_ 102.0, two methyls at δc 20.1 (Me-4) and 20.1 (Me-5). The ^1^H-^1^H COSY spectrum of compound **1** showed correlations in H2-H3-H4 and H5, H1′-H2′-H3′-H4′-H5′-H6′ and H5″-H6″, supporting the presence of a secondary amide [10]. The HMBC correlations from H-2 to C-1, C-3, C-4 and C-5, H-1′ to C-1 and C-3′, H-2′ to C-4, H-3′ to C-1 and C-5, H-4′ to C-2′ and C-6′, H-5′ to C-3′ and C-1″, H-6′ to C-4′, C-2″ and C-6″, H-2″ to C-4′, C-4″ and C-6″, H-5″ to C-1″ and C-3″, H-6″ to C-6′, C-2″ and C4″, and OCH_2_O to C-3″ and 4″, which was verified by correlations observed in the ^1^H-^1^H COSY and HMBC spectra (Figure 3) Therefore, the structure of compound **1** was elucidated as the new amide and namely as piperenamide A (Figure 2).

Piperenamide B (**2**) was observed as a colorless amorphous solid, with its molecular composition established as C_17_H_21_NO_3_, based on HR-TOFMS. This showed a [M + H]^+^ ion peak at **m*/*z** 288.1567 (calcd. C_17_H_22_NO_3_ **m*/*z** 288.1521), requiring to eight degrees of unsaturation (Figure 2). The UV spectrum showed maximum absorption at 270 and 241 nm, indicating the presence of a benzene and conjugated alkene. The IR spectrum showed bands which were ascribed to an amine (ν_max_ 3235 cm^−1^), amide carbonyl (νmax 1690 cm^−1^), isolated double bond conjugated (ν_max_ 1610 and 1504 cm^−1^), and ether groups (ν_max_ 1241 cm^−1^). The NMR spectra of two was very similar to those of one (Table 3), except with lost of methylene at δ_H_ 2.66 (2H, t, 7.5 Hz, H-6′); δ_C_ 35.9, C-6′) and methylene at δ_H_ 2.44 (2H, dd, 7.2; 7.5 Hz, H-5′), while two, methylene at δ_H_ 2.39 (2H, d, 7.2 Hz, H-5′), indicating that 2 was a demethylene derivative of one. In the HMBC spectrum, methylene correlations from H-5′ to C-1″, C-2″ and C-6″, suggest methylene δ_H_ 2.39; δ_C_ 35.4, directly apply to benzene (Supplementary Materials). Therefore, leading to the structure of **2** had been elucidated as the new amide, namely as piperenamide B (Figure 2).

## 3. Materials and Methods

### 3.1. General Experimental Procedures

UV spectra was measured using a TECAN Infinite M200 pro, with MeOH. The IR spectra and mass spectra were recorded on a SHIMADZU IR Prestige-21 in KBr and Waters Xevo QTOF MS, respectively. Using an NMR JEOL ECZ-500 and Variant Unity INOVA-500 Spectrometer (Agilent Technologies, Santa Carla, CA, USA), the NMR data was recorded at 500 MHz for ^1^H and 125 MHz for ^13^C, using TMS as internal standard. Column chromatography was conducted on the silica gel 60 (70–230 and 230–400 mesh, Merck, Kenilworth, NJ, USA), after which TLC analysis was carried out on 60 GF_254_ (Merck, 0.25 mm) using various solvent systems, in order to detect spots by irradiating under ultraviolet-visible light (257 and 364 nm) and heating of silica gel plates, sprayed with H_2_SO_4_ in EtOH (10%).

### 3.2. Plant Material

The leaf of *P. betle* L. var. *nigra* were collected from Samarinda, East Kalimantan, Indonesia in June 2020. Futhermore, the plant was identified By Dr. Atik Retnowati a staff of the Bogoriense Herbarium, Bogor, Indonesia. Finally, a voucher specimen (No. 749/IPH.1.01/If.07/VII/2020) was deposited at the Herbarium.

### 3.3. Extraction and Isolation

Method for extraction and isolation was referred to supriatno et al. (2018) [24]. The dried ground leaf (473.21 g) of *P. betle* L. var *nigra* was extracted with ethanol 70% (14 L), at room temperature for 7 days. After removal of the solvent under vacuum, the viscous concentrated EtOH extract (14.61 g) was obtained. The EtOH extract (14.61 g) was fractionated by column chromatography on silica gel, using a gradient of n-hexane, EtOAc and MeOH (10% stepwise) resulting into eight fractions (A–H). Fraction D (2.32 g) was subjected to column chromatography on silica gel using n-hexane-CHCl_3_-EtOAc (5% stepwise), as eluting solvents to afford seven subfractions (D1–D7). Subfraction D3 (882.2 mg) was chromatographed on a column of silica gel, eluted with n-hexane: EtOAc (7:3), to give seven subfractions (D3A–D3G). Similarly, subfraction D3D (120.3 mg) was chromatographed on silica gel eluted with n-hexane: CHCl_3_: EtOAc (7:2:1), to give **1** (8.2 mg). Subfraction D3E (90.1 mg) was chromatographed on silica gel eluted with petroleum ether: CHCl_3_ (4.5:5.5), to give **2** (2.8 mg) [24].

#### 3.3.1. Piperenamide A (1)

Colorless amorphous solid; mp 185−190 °C; UV (MeOH) λmax (log ε) 270 (4.01) and 241 (3.91) nm; IR (KBr) vmax 3295, 1656, 1611, 1504, 1490, 1443, 1141 and 1030 cm^−1^; HR-TOFMS *m*/*z* 302.1751 [M + H]^+^, (calcd. C_18_H_23_NO_3_ *m*/*z* 301,1749); ^1^H-NMR (CDCl_3_, 500 MHz), see Table 1; ^13^C-NMR (CDCl_3_, 125 MHz), see Table 1.

#### 3.3.2. Piperenamide B (2)

Colorless amorphous solid; mp 199−211 °C; UV (MeOH) λ_max_ (log ε) 268 (3.91), and 219 (3.67) nm; IR (KBr) *v*_max_ 3235, 1690, 1610, 1504, 1480, 1443, 1141 and 1030 cm^−1^; HR-TOFMS **m*/*z** 288.1567 (calcd. C_17_H_22_NO_3_ **m*/*z** 288.1521) ^1^H-NMR (CDCl_3_, 500 MHz), see Table 1; ^13^C-NMR (CDCl_3_, 125 MHz), see Table 1.

### 3.4. Antimicrobial Activity

#### 3.4.1. Material

Liquid media for this study were nutrient broth (NB; Oxoid, Hampshire, UK) and tryptic soy broth (TSB; Merck). Solid media were Mueller Hinton Agar (MHA; Oxoid) and Tryptic soy agar (TSA; Merck)

#### 3.4.2. Method

The antimicrobial activity of *P. betle* extract was performed by well diffusion method against two oral pathogenic bacteria (*Streptococcus mutans, Streptococcus sanguinis*) and opportunistic pathogenic yeast (*Candida albicans*). The inoculum (bacterial and yeast suspension) was prepared from a 24-h culture on NB for bacterial suspension and on TSB for yeast suspension. Each of inoculum was diluted with sterile physiological solution (0.9%) to 108 CFU/mL (McFarland standard 0.5). 20 mL of each agar media were melted (NA media for bacterial and TSA media for yeast), cooled to 50 °C and then inoculated with 0.2 mL of the microbial suspension. The inoculated agar was poured into sterile petri dish, and then allowed to cool down on a leveled surface. Once the media had compacted, two wells were cut out of the agar, each 6 mm in diameter. Then, 30 µL of the extract sample (concentration of 0.5 and 1%) were added into each well and incubated for 24 h at 36 °C ± 1°C under aerobic condition. Inhibition of microbial growth was measured in mm using SCAN 500^®^ tools. Tests were performed in duplicate.

## 4. Conclusions

The presents study showed antimicrobial activity from crude ethanol extract of leaves *P. betle* L. var. *nigra*. The first report of two new amides derivatives, piperenamide A (**1**) and piperenamide B (**2**) in *P. betle* L. var. *nigra*.

## Figures and Tables

**Table 1:** Growth inhibition diameters of *Candida albicans*, *Streptococcus mutans*, and *Streptococcus sanguinis* by *Piper betle* var. *nigra* extract.

Microbial	Inhibition Diameters ± SD (mm)	
	Extract Concentration of 0.5%	Extract Concentration of 1%
*Candida albicans*	16.7 ± 0.8	18.1 ± 0.7
*Streptococcus mutans*	18.2 ± 1.8	19.6 ± 1.9
*Streptococcus sanguinis*	9.9 ± 1.8	12.3 ± 1.1

**Table 2:** Growth inhibition diameters of *Candida albicans, Streptococcus mutans,* and *Streptococcus sanguinis* by Piper betle var. *nigra* isolate piperamide A and B.

Microbial	Inhibition Diameters ± SD (mm)	
	Piperamide A	Piperamide B
	Concentration of 0.02%	Concentration of 0.02%
*Candida albicans*	-	-
*Streptococcus mutans*	-	-
*Streptococcus sanguinis*	-	-

**Table 3:** NMR data compound **1**–**2** (500 MHz for ^1^H dan 125 MHz for ^13^C).

Position Carbon	1		2	
	^13^C-NMR	^1^H-NMR	^13^C-NMR	^1^H-NMR
	δC (mult.)	δC [(ΣH, mult, J(Hz)]	δC (ppm)	δC [(ΣH, mult, J(Hz)]
**1**	169.1 (s)	-	168.4 (s)	-
**2**	48.0 (t)	3.06 (2H, d, 6.7)	47.3 (t)	3.10 (2H, d, 6.7)
**3**	29.7 (d)	1.78 (1H, qt, 6.7)	28.9 (d)	1.82 (1H, qt, 6.7)
**4**	20.1 (q)	0.90 (3H, d, 6.7)	20.1 (q)	0.94 (3H, d, 6.7)
**5**	20.1 (q)	0.90 (3H, d, 6.7)	20.1 (q)	0.94 (3H, d, 6.7)
**1′**	123.3 (d)	5.92 (1H, dd, 4.6; 15.1)	122.6(d)	5.96 (1H, dd, 4.6; 15.1)
**2′**	141.9 (d)	7.02 (1H, dd, 10.6; 15.1)	141.2 (d)	7.12 (1H, dd, 10.6; 15.1)
**3′**	142.8 (d)	6.09 (1H, dd, 10.6; 15.2)	142.0 (d)	6.12 (1H, dd, 10.6; 15.2)
**4′**	130.2 (d)	6.18 (1H, dd, 7.2; 15.2)	129.6 (d)	6.19 (1H, dd, 7.2; 15.2)
**5′**	36.1 (t)	2.44 (2H, dd, 7.2; 7.5)	35.4 (t)	2.39 (2H, d, 7.2)
**6′**	35.9 (t)	2.66 (2H, t, 7.5)	-	-
**1″**	136.5 (s)	-	135.8 (s)	-
**2″**	109.7 (d)	6.69 (1H, d, 1.5)	109.8 (d)	6.73 (1H, d, 1.5)
**3″**	149.0 (s)	-	148.3 (s)	-
**4″**	147.9 (s)	-	146.4 (s)	-
**5″**	108.8 (t)	6.69 (1H, d, 7.9)	108.2 (t)	6.73 (1H, d, 7.9)
**6″**	122.3 (d)	6.63 (1H, dd, 1.5, 7.9)	121.6 (d)	6.68 (1H, dd, 1.5, 7.9)
**OCH_2_O**	102.0 (t)	5.88 (2H, s)	101.2 (t)	5.91 (2H, s)
**NH**	-	4.62 (1H, br.s)	-	4.65 (1H, br.s)

## Data Availability

The data presented in this study are available on request from the corresponding author.

## Supplementary Materials

The following are available online. Figure S1: 1H-NMR Spectra of (1), Figure S2: 13C-NMR Spectra of (1), Figure S3: DEPT-135° Spectrum of (1), Figure S4: HMQC Spectrum of (1), Figure S5: HMBC Spectrum of (1), Figure S6: 1H-1H-COSY Spectra of (1), Figure S7: TOF MS Spectra of (1), Figure S8: 1H-NMR Spectra of (2), Figure S9: 13C-NMR Spectrum of (2), Figure S10: DEPT-135° Spectrum of (2), Figure S11: HMQC Spectrum of (2), Figure S12: HMBC Spectrum of (2), Figure S13: 1H-1H-COSY Spectra of (2), Figure S14: TOF MS Spectra of (2)
