# Supplementary material for: Novel Amides Derivative with Antimicrobial Activity of Piper betle var. nigra Leaves from Indonesia
Source: Molecules. 2021 Jan 11;26(2):335. doi: 10.3390/molecules26020335 (PMC7826617; doi:10.3390/molecules26020335)
Supplement: Supplementary file 1 [file molecules-26-00335-s001.pdf]

molecules

Article

Novel Amides Derivative with Antimicrobial Activity of *Piper Betle* var. *nigra* Leaves from Indonesia

Fajar Prasetya [1], Supriatno Salam [2], Agung Rahmadani [1,2,3], Kansy Haikal [2], Lizma Febrina [1], Hady Anshory [4], Muhammad Arifuddin [1], Vita Olivia Siregar [1], Angga Cipta Narsa [1], Herman Herman [1], Islamudin Ahmad [1], Niken Indriyanti [1], Arsyik Ibrahim [1], Rolan Rusli [1], Laode Rijai [1] and Hadi Kuncoro [1,*]

[1] Faculty of Pharmacy, Universitas Mulawarman, Samarinda, Kalimantan Timur 75123, Indonesia; fajarprasetya@farmasi.unmul.ac.id (F.P.); agungchempharm8794@gmail.com (A.R.); lizmafebrina@farmasi.unmul.ac.id (L.F.); marifuddin@farmasi.unmul.ac.id (M.A.); vitaoliviasiregar@farmasi.unmul.ac.id (V.O.S.); angga@farmasi.unmul.ac.id (A.C.N.); herman@farmasi.unmul.ac.id (H.H.); islamudinahmad@farmasi.unmul.ac.id (I.A.); nikenindriyanti@farmasi.unmul.ac.id (N.I.); arsyik@farmasi.unmul.ac.id (A.I.); rolan@farmasi.unmul.ac.id (R.R.); laoderijai@farmasi.unmul.ac.id (L.R.).

[2] Department of Chemistry, Faculty of Mathematics and Natural Sciences, Universitas Padjadjaran, Jatinangor 45363, Indonesia; supriatno.salam@gmail.com (S.S.); kansyhaikal@unpad.ac.id (K.H.)

[3] Departement of Chemistry Education, Faculty of Teaching and Education, Mulawarman University, Samarinda 75123, Kalimantan Timur, Indonesia

[4] Departement of Pharmacy, Faculty of Mathematics and Natural Sciences, Islamic University of Indonesia, Jogjakarta 55584, Indonesia; hadyanshory@uii.ac.id

* Correspondence: hadikuncoro@farmasi.unmul.ac.id; Tel.: +62-541-739-491; Cell Phone: +62-82-158-220-088

Table of Contents	Page
**Figure S1.** ^{1}H-NMR Spectra of (**1**) (500 MHz in CD_3OD)	3
**Figure S2.** ^{13}C-NMR Spectrum of (**1**) (125 MHz in CD_3OD)	4
**Figure S3.** DEPT-135° Spectrum of (**1**) (135 MHz in CD_3OD).	5
**Figure S4.** HMQC Spectrum of (**1**).	6
**Figure S5.** HMBC Spectrum of (**1**).	7
**Figure S6.** ^{1}H-^{1}H-COSY Spectra of (**1**).	8
**Figure S7**. TOF MS Spectra of (**1**)	9
**Figure S8**. ^{1}H-NMR Spectra of (**2**) (500 MHz in CD_3OD)	10
**Figure S9**. ^{13}C-NMR Spectrum of (**2**) (125 MHz in CD_3OD)	11
**Figure S10.** DEPT-135° Spectrum of (**2**) (135 MHz in CD_3OD).	12
**Figure S11.** HMQC Spectrum of (**2**).	13
**Figure S12.** HMBC Spectrum of (**2**).	14
**Figure S13.** ^{1}H-^{1}H-COSY Spectra of (**2**).	15
**Figure S14**. TOF MS Spectra of (**2**)	16

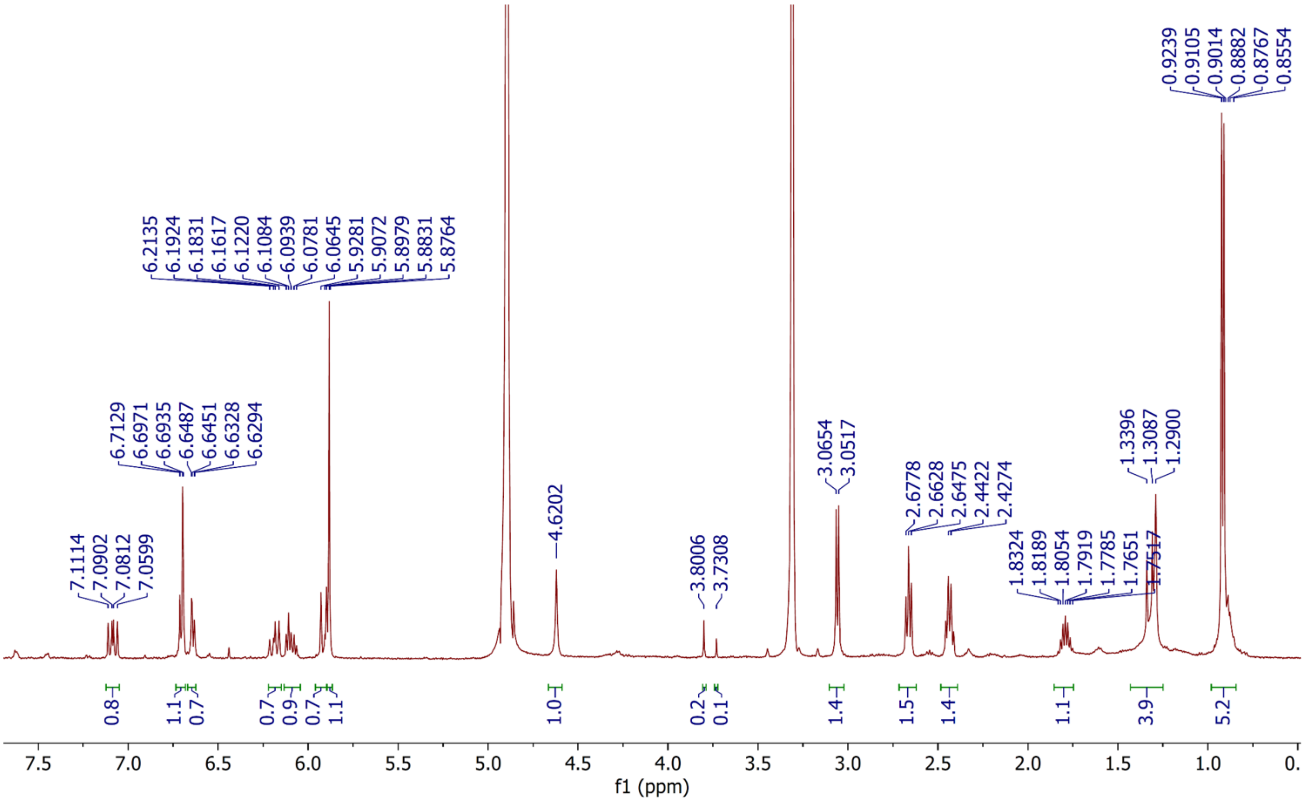

Figure S1. ^{1}H-NMR Spectra of (**1**) (500 MHz in CD_3OD).

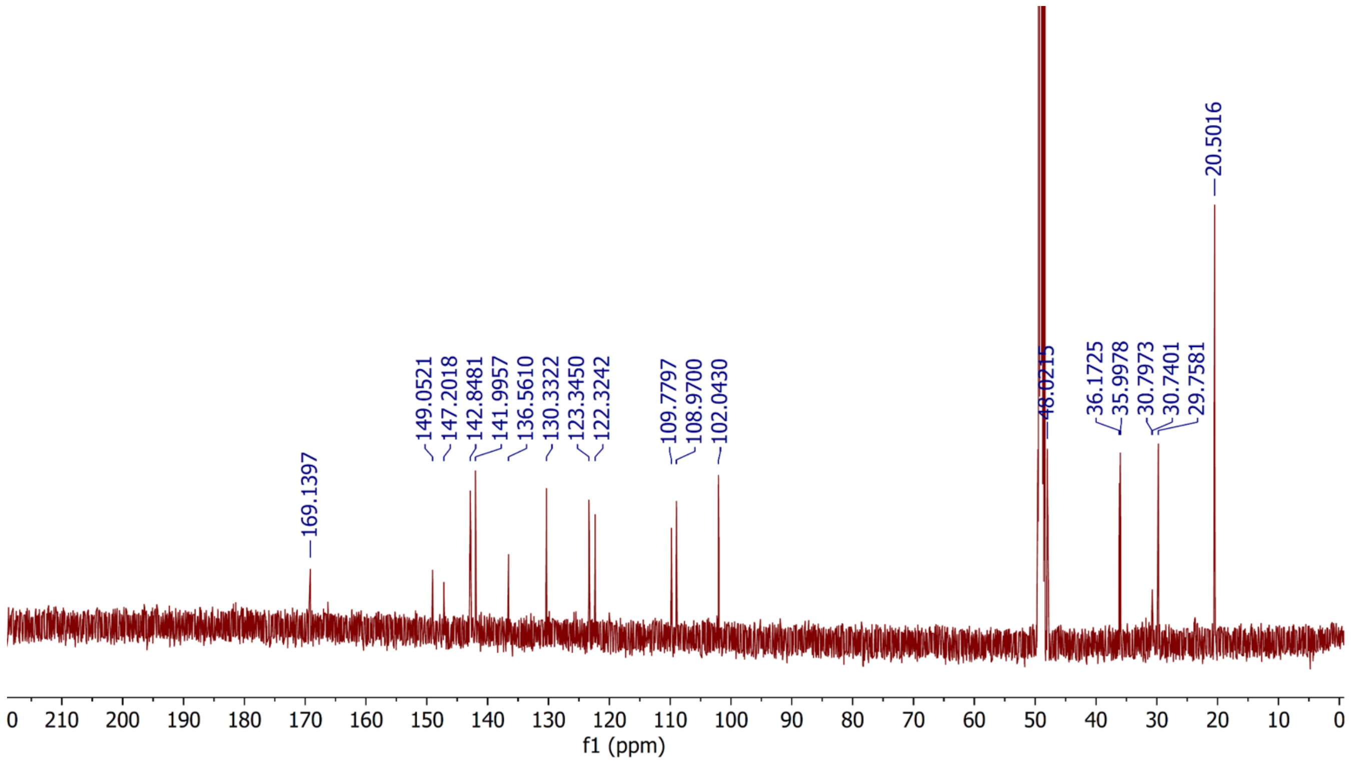

Figure S2. ^{13}C-NMR Spectrum of (**1**) (125 MHz in CD_3OD).

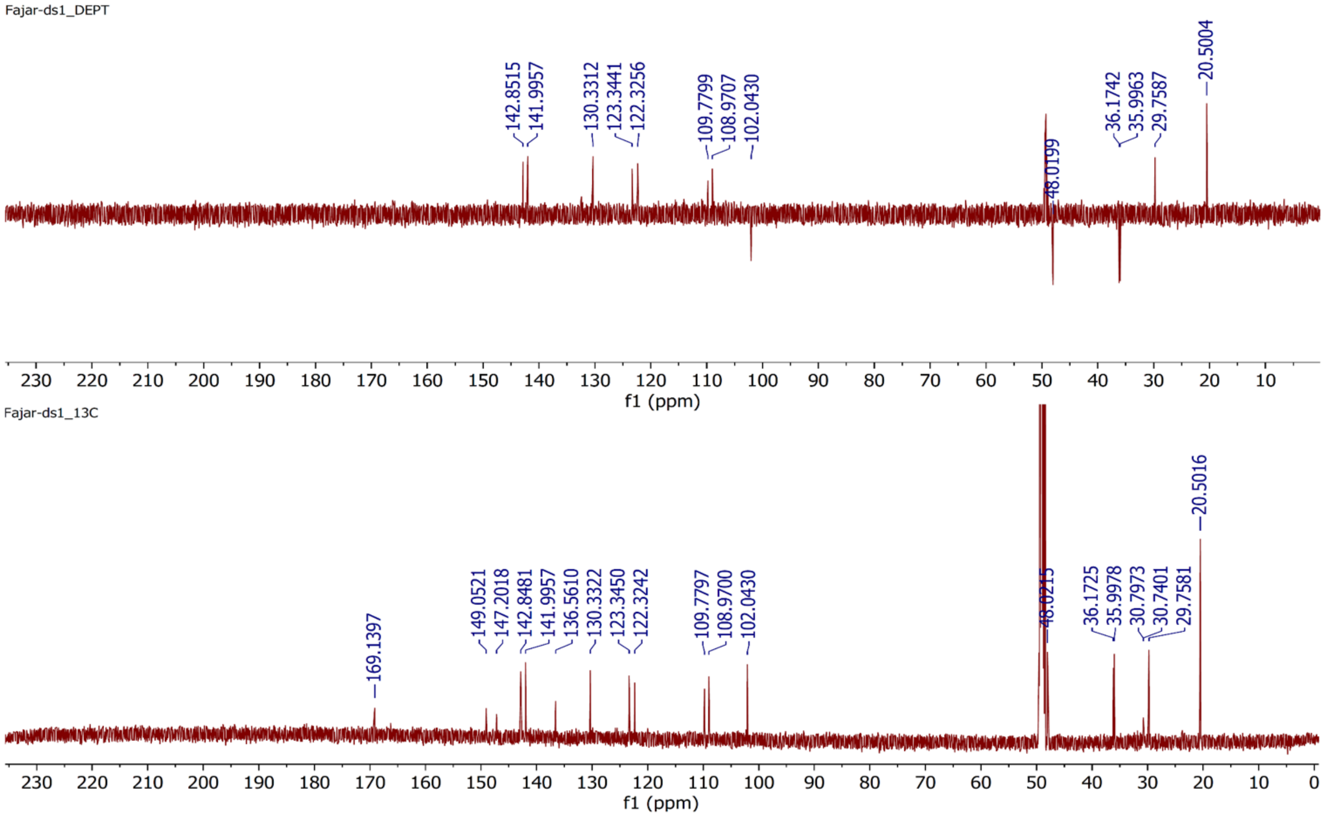

Figure S3. DEPT-135° Spectrum of (**1**) (135 MHz in CD_3OD).

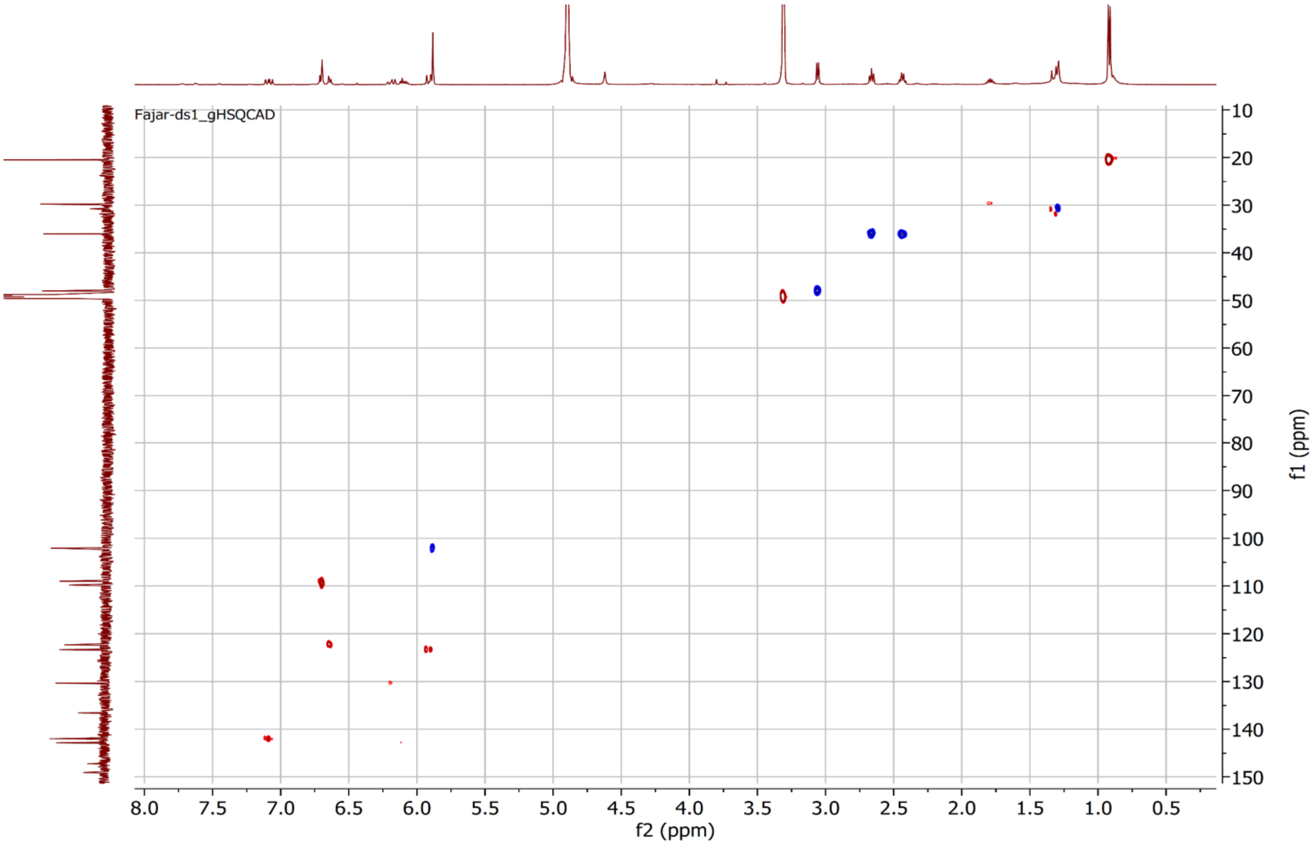

Figure S4. HMQC Spectrum of (**1**).

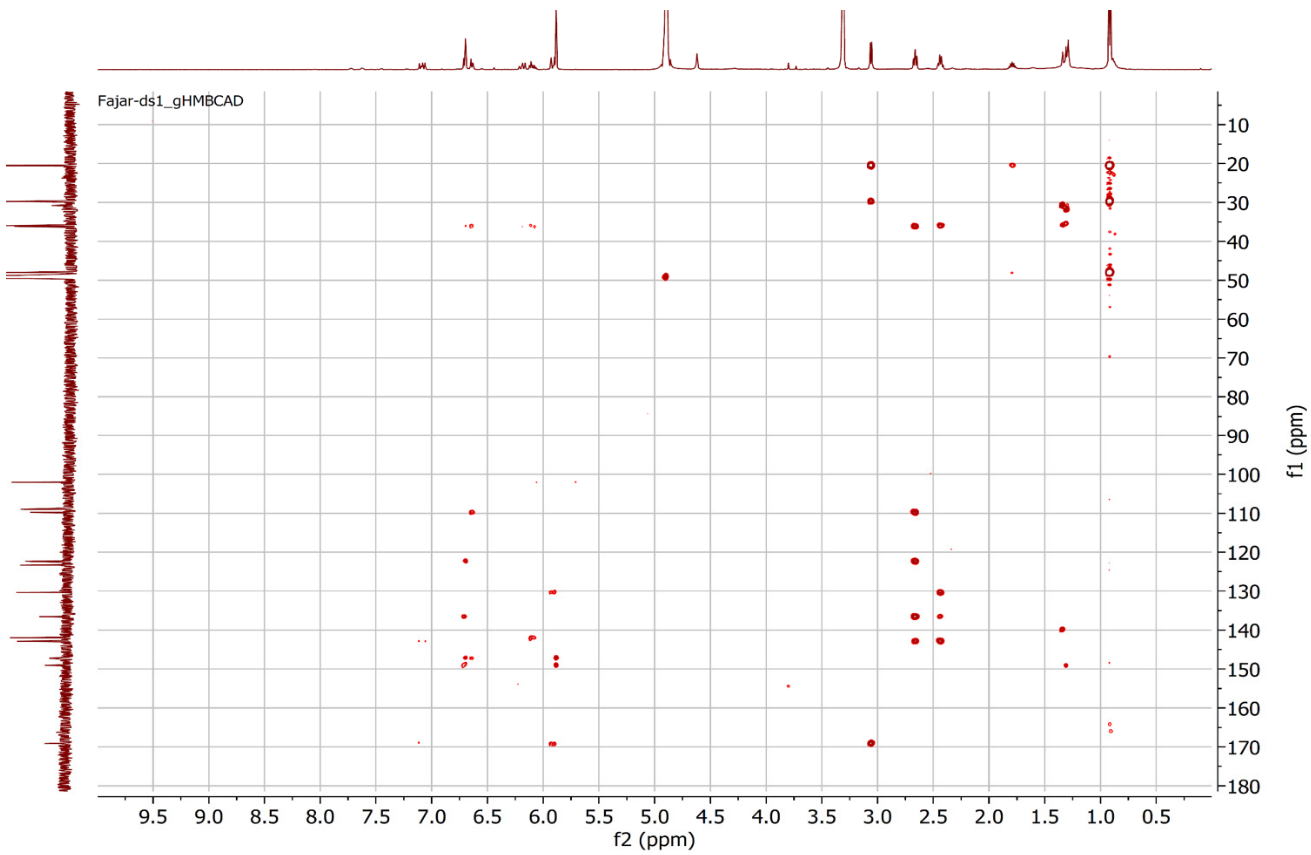

Figure S5. HMBC Spectrum of (**1**).

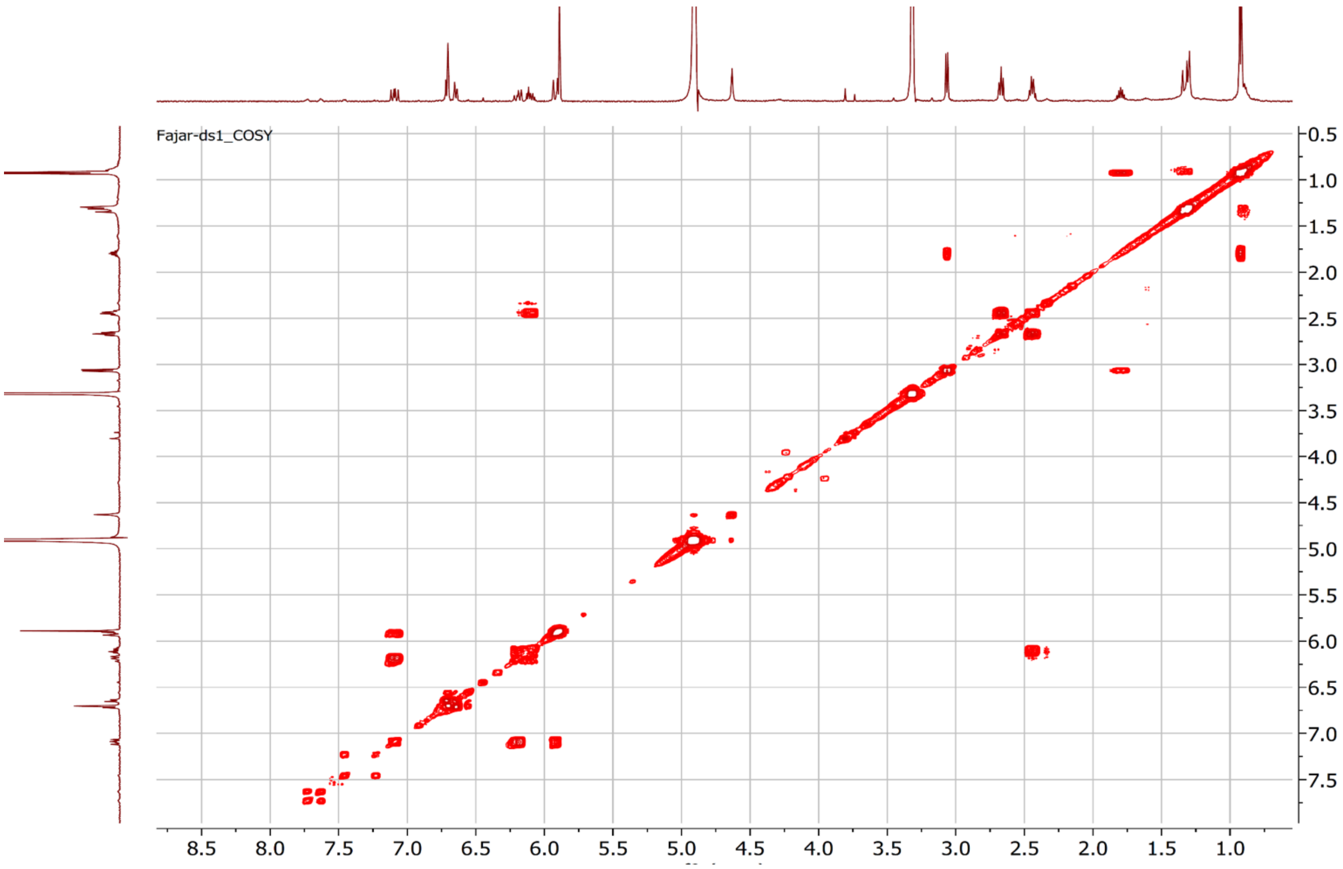

Figure S6. ^{1}H-^{1}H-COSY Spectra of (**1**).

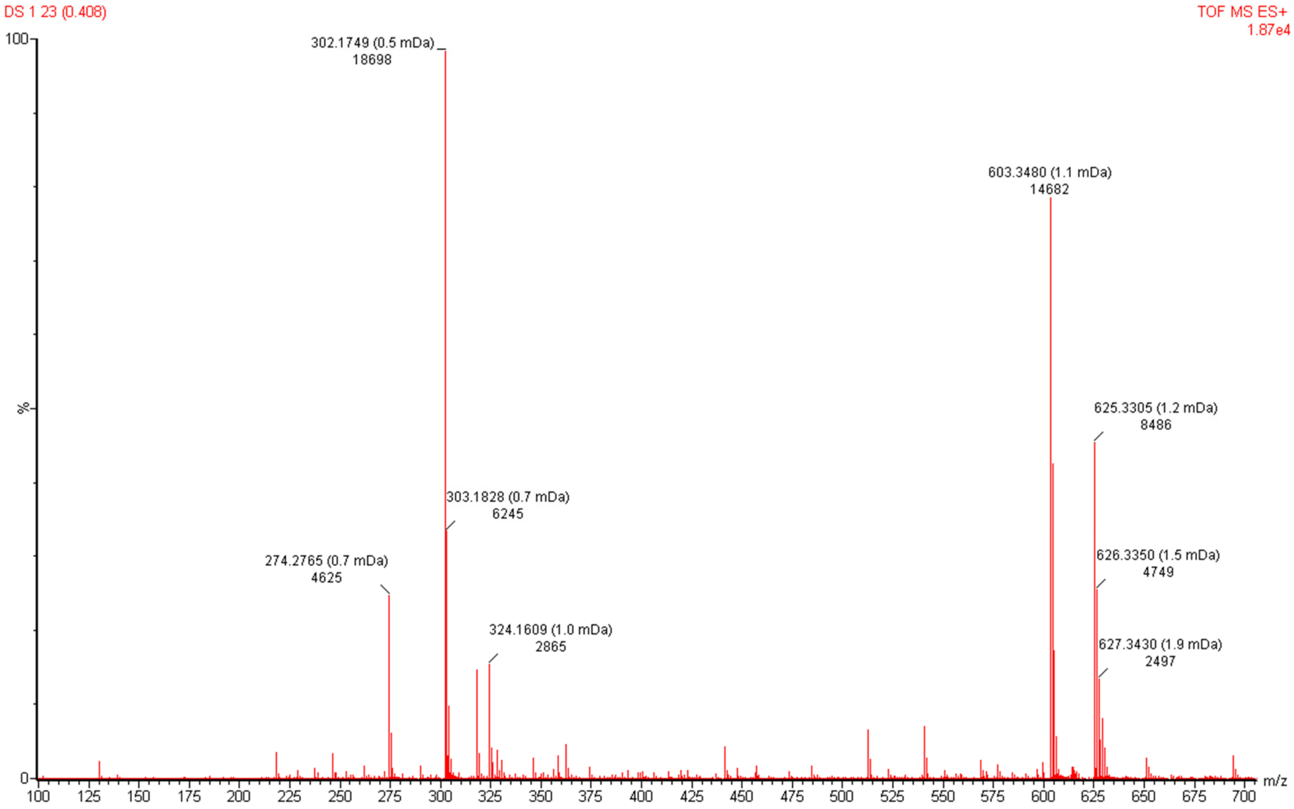

Figure 7. TOF MS Spectra of (**1**).

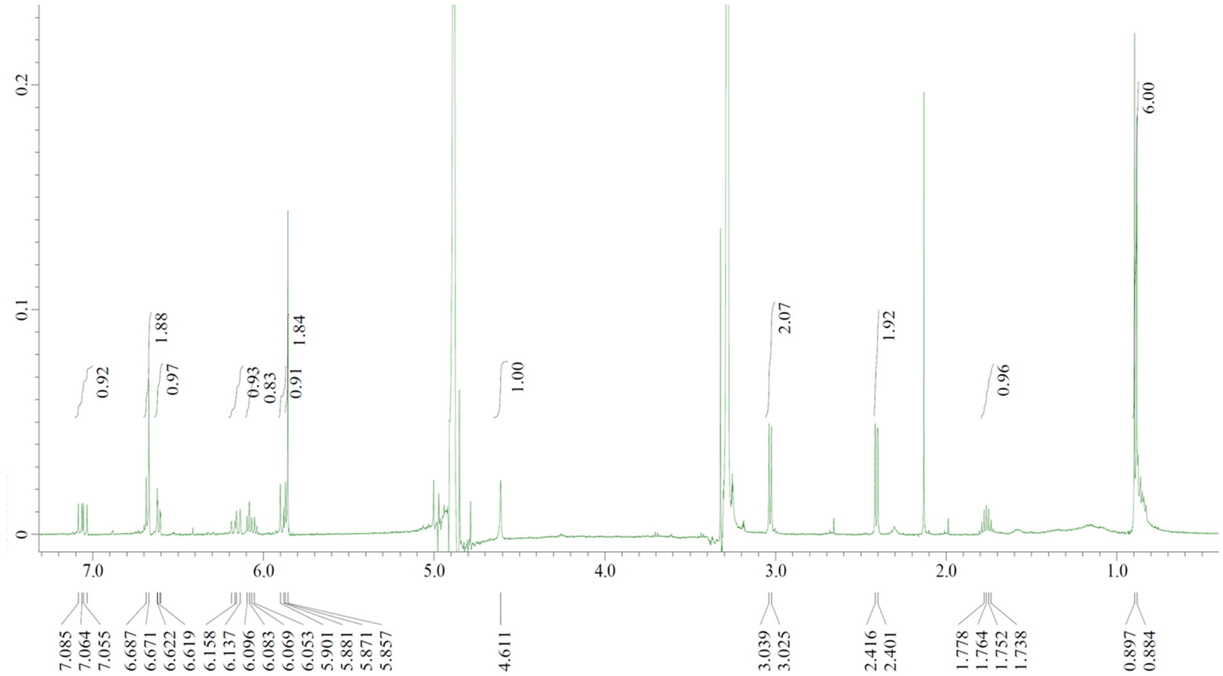

Figure S8. ^{1}H-NMR Spectra of (**2**) (500 MHz in CD_3OD).

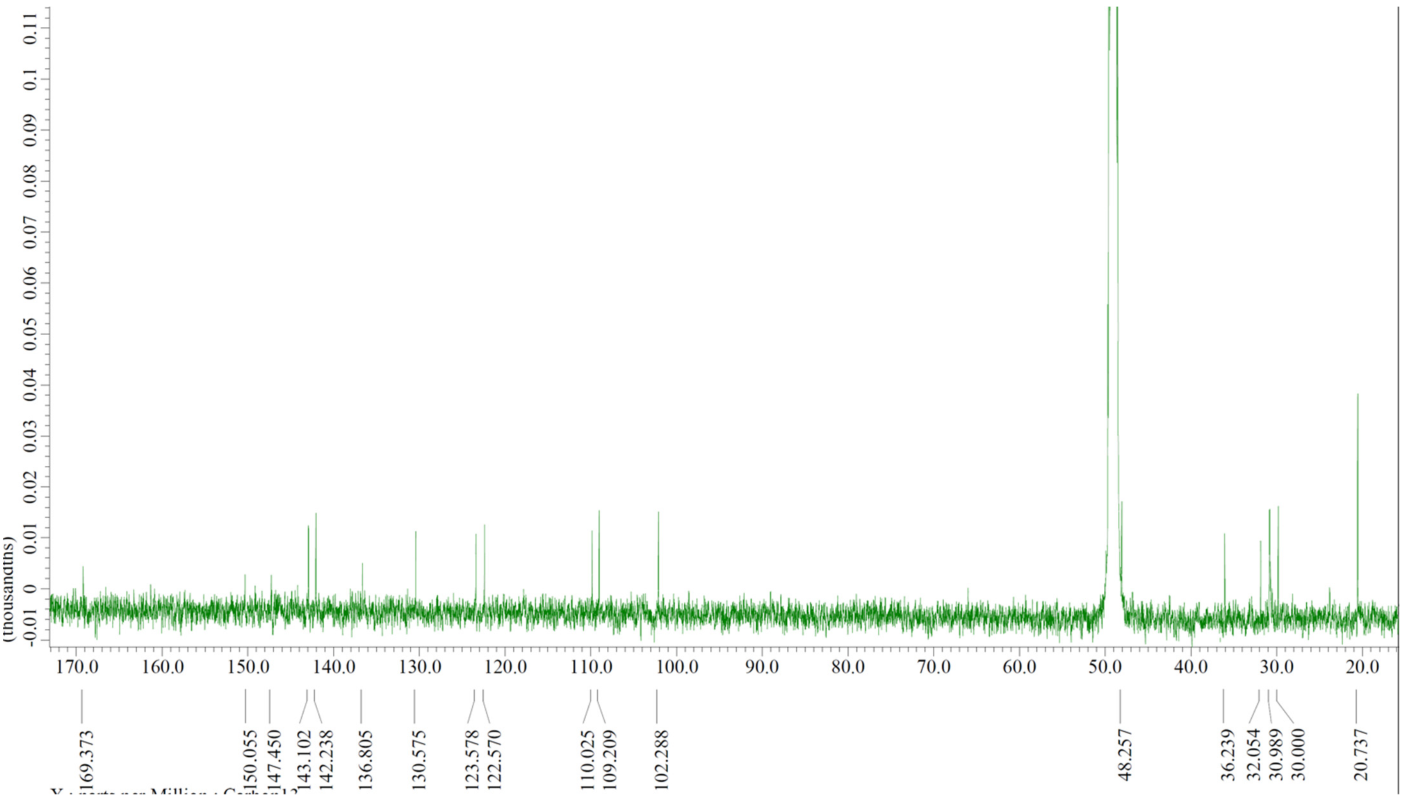

Figure S9. ^{13}C-NMR Spectrum of (**2**) (125 MHz in CD_3OD).

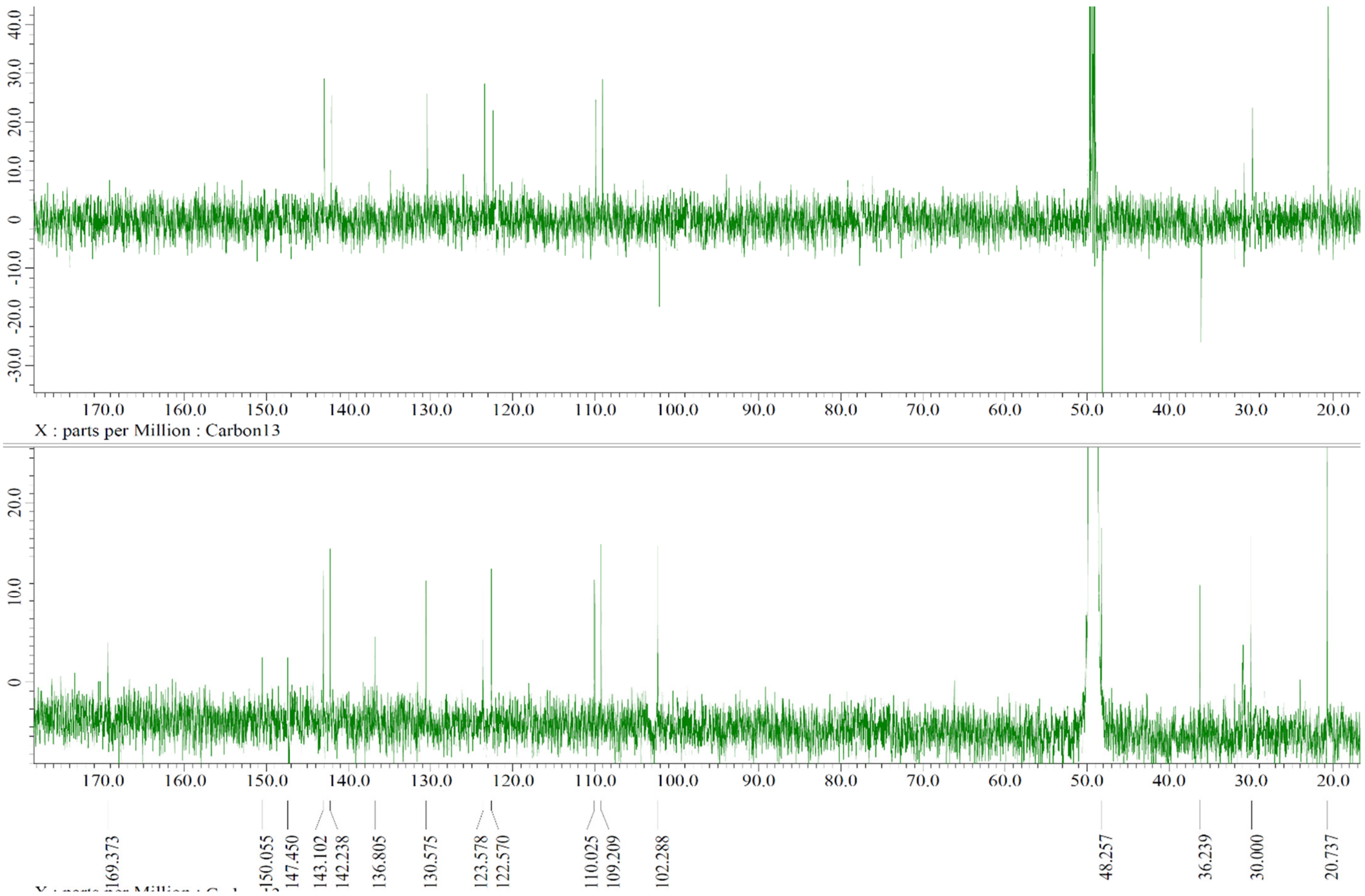

Figure S10. DEPT-135° Spectrum of (2) (135 MHz in CD_3OD).

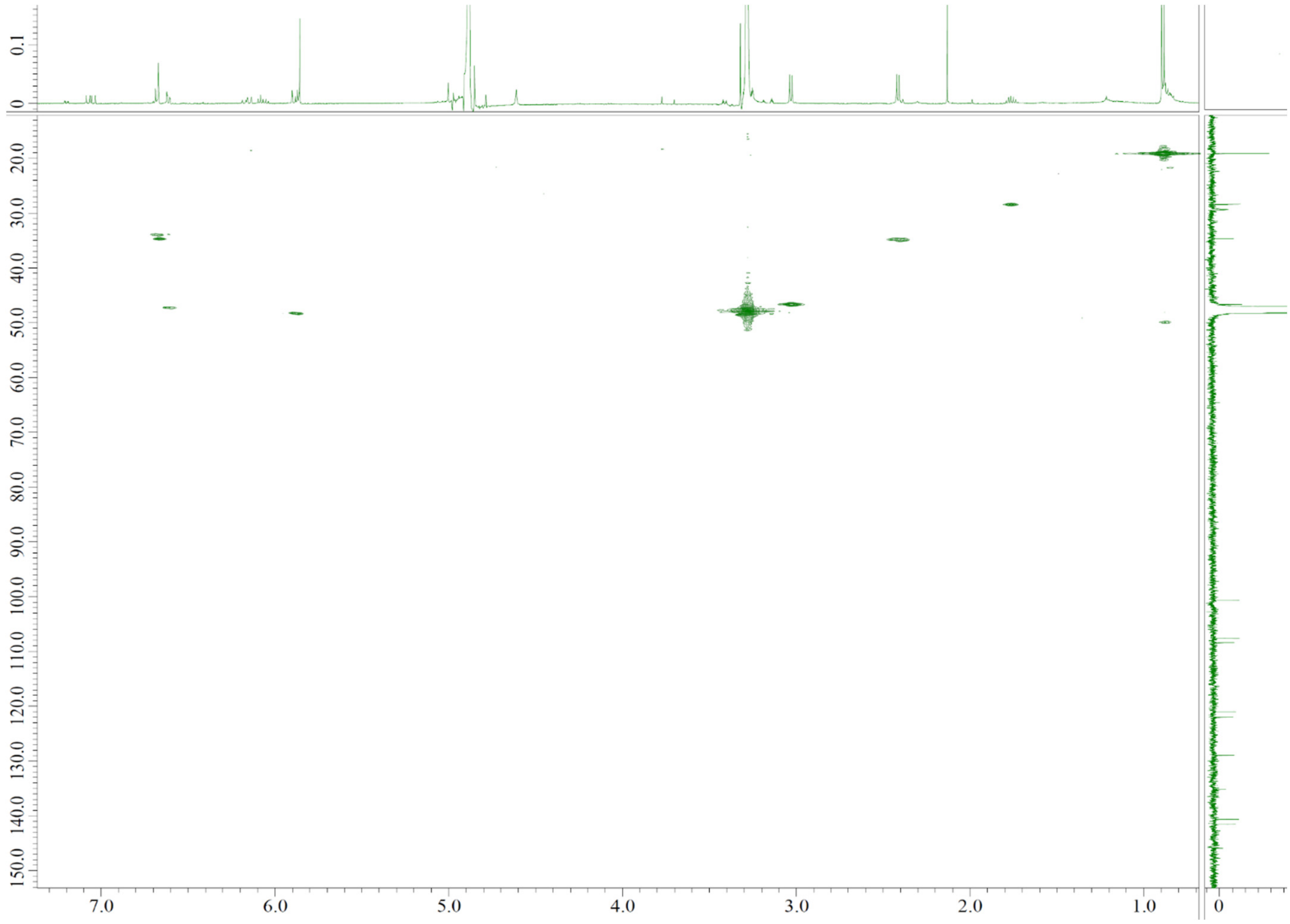

Figure S11. HMQC Spectrum of (**2**).

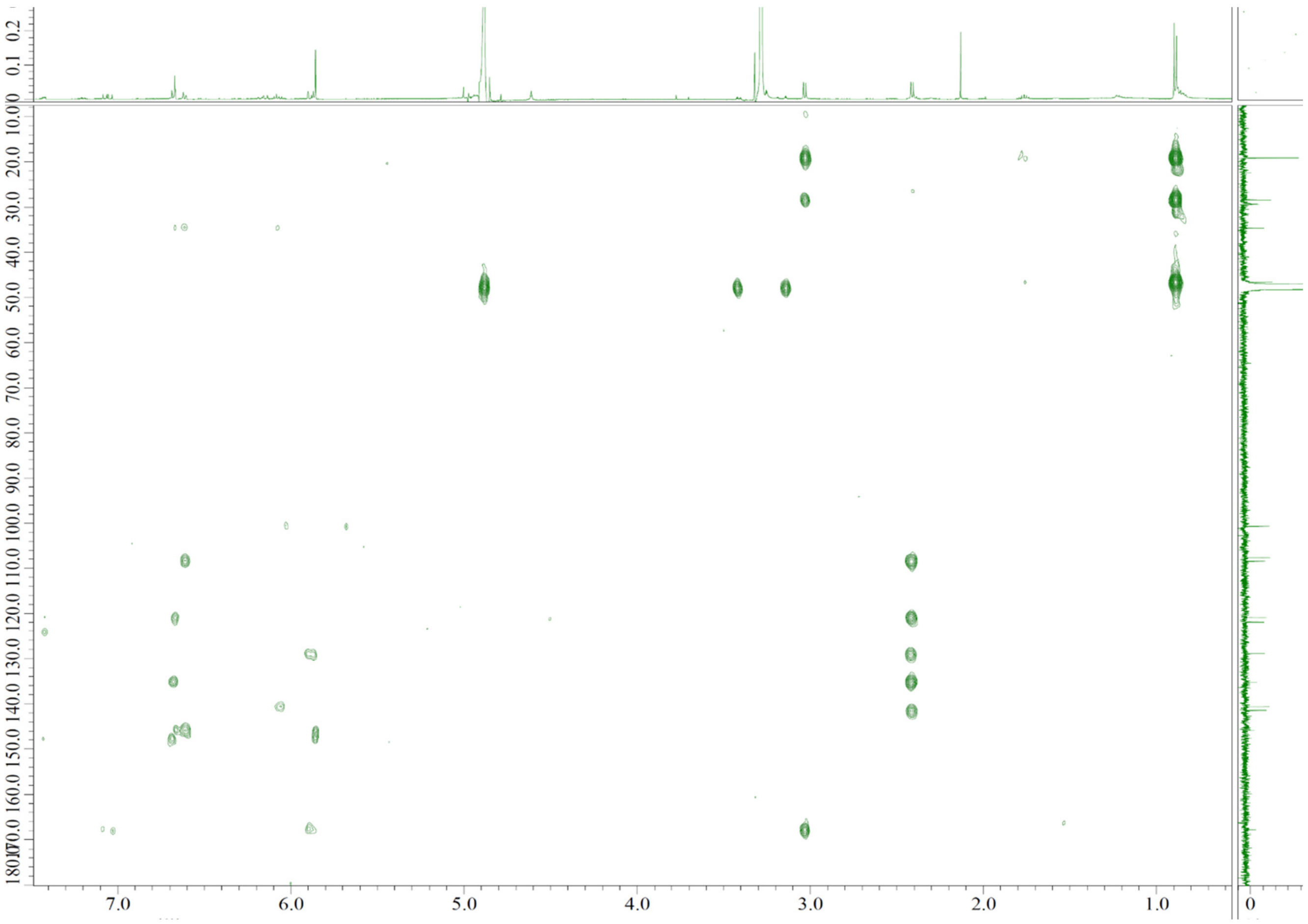

Figure S12. HMBC Spectrum of (**2**).

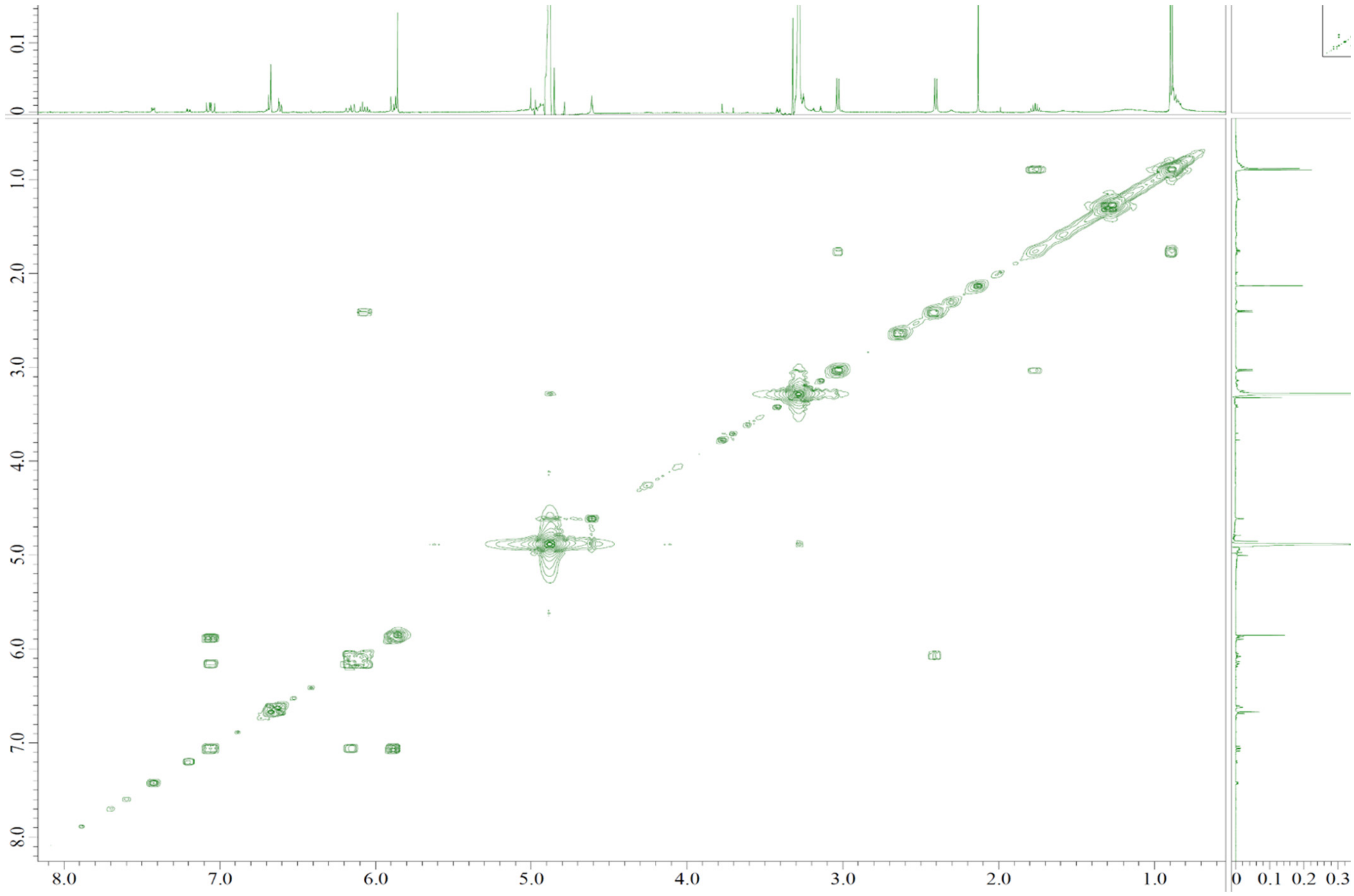

Figure S13. ^{1}H-^{1}H-COSY Spectra of (**2**).

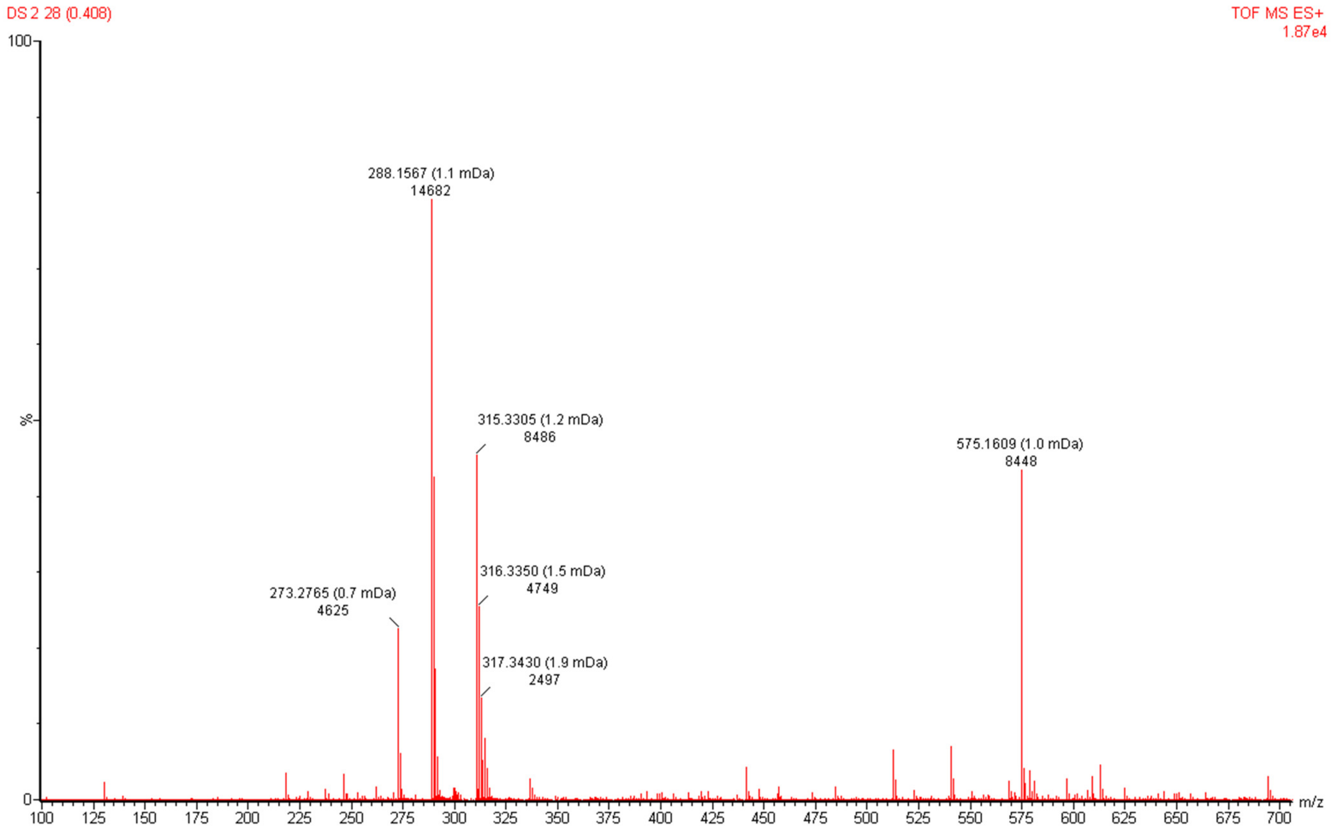

Figure S14. TOF MS Spectra of (**2**).
